# Oncotype DX tumor gene expression profiling in stage II colon cancer: Application: Prognostic, risk prediction

**DOI:** 10.1371/currents.RRN1177

**Published:** 2010-09-02

**Authors:** Elizabeth M. Webber, Jennifer S. Lin, Evelyn P. Whitlock

**Affiliations:** Kaiser Permanente Center for Health Research

## Abstract

Overall five-year survival for patients with stage-II colon cancer averages 75% after surgery alone. However, some of these patients have poorer outcomes, similar to patients with stage-III disease. The proposed use of the Oncotype DX assay is to improve risk stratification for recurrence in stage-II colon cancer.

## **Clinical Scenario**

Onco*type* DX is used for profiling tumor gene expression in patients with Stage-II colon cancer to predict recurrence risk and inform treatment decisions following surgery.

## **Test Development**

Four studies (n=1851) reported the results of the initial development of the Onco*type* Dx assay in colon cancer. These studies were conducted by the manufacturer in partnership with the National Surgical Adjuvant Breast and Bowel Project and the Cleveland Clinic.[1] [2] [3] During initial development the assay comprised an 18-gene panel that included 7 genes for relapse-free survival prognosis in colon cancer to yield a prognostic recurrence score (RS), 6 genes to predict response to 5-fluorouracil/leucovorin (5FU/LV) chemotherapy to yield a predictive treatment score (TS), and 5 reference genes. Further development of the 12-gene RS in these four studies found that there were no apparent differences in gene expression patterns between stage-II and stage-III colon cancer patients.[4]

Subsequent validation of the 18 gene panel found that the RS score was a valid predictor of relapse-free survival. The TS score, however, was not found to be a valid predictor of treatment response.[5] [6] Because of this result, these 6 predictive TS genes were not included in the test currently marketed by Genomic Health.

## **Test Description**

Onco*type* DX is a quantitative multi-gene, real-time polymerase chain reaction (RT-PCR) assay that measures gene expression in paraffin-embedded tumor tissues.[5] The Onco*type* DX assay that Genomic Health plans to market in 2010 will include 7 genes for relapse-free survival prognosis and 5 reference genes and yields a prognostic recurrence score (RS).[6]

## **Public Health Importance**

Colorectal cancer is the third most common non-dermatological cancer in the United States and is the second leading cause of cancer-related death in the United States. The American Cancer Society estimates that 106,100 new cases of colon cancer (52,010 in men and 54,090 in women) were diagnosed in 2009.[7] [8] Ongoing controversy exists as to whether adjuvant chemotherapy should be advised for patients with stage-II colon cancer.[9] Identification of patients at higher risk of recurrence may help to inform decisions surrounding the use of adjuvant chemotherapy to potentially improve prognosis after surgery. 

## **Published Reviews, Recommendations and Guidelines**

**Systematic evidence reviews**

None identified

**Recommendations by independent group**

None identified

**Guidelines by professional groups**

None identified

## **Evidence Overview**

***Analytic Validity:*** Test accuracy and reliability in measuring differences in expression of relevant genes (analytic sensitivity and specificity).

- We identified no reports of analytic sensitivity or specificity for this assay. The majority of reports on the development of this assay have been published as meeting abstracts that do not include information related to analytic validity.
- We identified only one derivation study conducted on an initial set of 761 candidate genes, in which the authors state they developed a multi-analyte gene expression platform for biomarker discovery that maintains good test performance; however, the authors reported no specific analytic sensitivity or specificity calculations.[1]

***Clinical Validity:*** Test accuracy and reliability in predicting colon cancer recurrence (predictive value).

- One meeting abstract from 2009 reported on the evaluation of the clinical validity of the developmental 18-gene panel in the QUASAR clinical trial:
  - In a sample of 725 patients (out of a total of 1622 patients) who received adjuvant 5FU/LV chemotherapy, the treatment score (TS) did not predict benefit from chemotherapy (p=0.19).[5] [10]
  - In a sample of 711 patients (out of a total of 1617 patients) who were randomized to observation, the recurrence score (RS) was validated as a predictor of relapse-free progression and overall survival independent of known risk factors of recurrence (i.e., mismatch repair status, T stage, lymph nodes examined, grade, and lymphovascular invasion).[5] [10]
    - It was noted in a subsequent editorial (but not in the meeting abstract) that the average risk of recurrence at 3 years among QUASAR patients who did not undergo further treatment after cancer surgery was 12% for those the assay classified as low-risk, 18% for intermediate-risk, and 22% for the high-risk group.[7] [10]

- Another meeting abstract presented in 2010 reported the correlation of number of lymph nodes and the current 12-gene assay for colon cancer in the same validation sample from the QUASAR clinical trial:[11]
  - In a sample of 657 of 711 patients for whom data was available, the number of lymph nodes examined (current ASCO/NCCN quality measures recommend a minimum of at least 12 nodes[12]) and RS were both independent predictors of recurrence in stage-II colon cancer following surgery. Similar results were observed in models incorporating mismatch repair status and T stage. Because of these results, the authors suggest that both of these factors should be considered when assessing individual recurrence risk.[11]
    ***Clinical Utility:*** Net benefit of test in improving health outcomes.  
  - To date, no prospective studies have been conducted to establish the clinical utility of the Onco*type* DX colon cancer assay
   

Last updated: April 26, 2010

## **Limitations**

Overall, we found very little evidence, most identified studies related to the Onco*type* DX assay for colorectal cancer were development or initial validation studies. Most studies were also presented as meeting abstracts and not as complete publications, including the only two studies related the clinical validity. Full critical appraisal and confirmation of reported results are not possible without more details on these studies. The validation studies represent retrospective analyses on only a subset of the patients in the prospectively designed QUASAR trial. It is not clear if these samples represent the full spectrum of patients or a specially selected group that may over-estimate the assays performance. In addition, risk prediction was calculated as relative estimates between low-, intermediate, and high-risk categories, with a fairly narrow range across groups. The lack of a calculated absolute risk may lead to some difficulty in implementing this assay into clinical practice decisions and determining its true benefit. Further, no net benefit can be determined from validations studies that consider test performance only. 

## **Conclusions**

There is currently not enough evidence for a full evaluation of this assay. Although Genomic Health launched the Onco*type* Dx colon cancer assay worldwide in January 2010, additional research is clearly needed before the value of this assay for clinical practice can be determined. The manufacturer has indicated that their reference laboratory will perform the Onco*type* Dx colon cancer assay. At this point FDA approval will not be required for this assay because the assay will be performed in house by the Genomic Health commercial laboratory that is regulated and certified under the Clinical Laboratory Improvement Amendments (CLIA).[7]

## **Links**

- http://www.oncotypedx.com/COLON/index.aspx
- For recent additions to the literature, see Pubmed special query
- U.S. Food and Drug Administration: Search FDA 510(k) database

## Acknowledgments

We thank Smyth Lai for her assistance in searching for literature on this topic and Marta Gwinn and the CDC National Office of Public Health Genomics for their assistance in developing this review.  

## Funding information

This review was conducted as part of the evidence synthesis component (Project 1) of the Comparative Effectiveness Research in Genetics (CERGEN) in Colorectal Cancer, NCI RC 2-CA148471.  

## Competing interests

The authors have declared that no competing interests exist.
